# Where Are They Now? The University of Michigan Medical School Doctors of Tomorrow Pathway Program

**DOI:** 10.1007/s40670-026-02769-6

**Published:** 2026-06-06

**Authors:** Rico Ozuna-Harrison, Brandon Lu, Diamond Buchanan, Naomi Parker, Kristian Bennett, Murshed Ahmed, Serena Bidwell, Darrell Tubbs, Julie Evans, Jonathan F. Finks, Gurjit Sandhu

**Affiliations:** Department of Surgery, University of Michigan Medical School, 1500 E. Medical Center Dr., Ann Arbor, MI 48109 USA

**Keywords:** Matriculation, Medical students, Pathways, High school, Pipeline program

## Abstract

**Introduction:**

Pathway programs aim to increase diversity in medicine by providing exposure, opportunities, and mentorship for underrepresented minority (URiM) students. While many studies focus on undergraduate-level pathway programs, few examine high school students’ career trajectories. Doctors of Tomorrow (DoT) is a high school pipeline program at the University of Michigan that partners with Detroit high schools. This study evaluates the outcomes of this program and the additional needs for achieving its goal of improving diversity in the medical workforce.

**Methods:**

A cross-sectional survey was conducted on DoT alumni from the classes of 2016 to 2021 to assess education and career trajectories. Quantitative data and qualitative data were analyzed to compare demographic information, educational trajectories, career aspirations as well as common barriers and facilitators that participants encountered on their paths toward healthcare careers.

**Results:**

A survey of 75 DoT alumni (41% response rate) revealed that 98% attended college, 52% pursued pre-medicine tracks, and 57% aimed to become physicians. Key themes included awareness, preparedness, and social capital. Financial challenges and a lack of social support were key barriers. Mentorship and early exposure to healthcare facilitated persistence.

**Conclusions:**

DoT shows high college enrollment and interest in medical careers among alumni, but financial and social barriers persist. Despite the program’s success, few alumni have entered medical school, highlighting the need for support systems such as sustained mentorship, financial assistance, and institutional guidance. Enhancing these types of support for students is crucial to improving URiM representation in medicine and ultimately health outcomes for underserved communities.

## Introduction

Pathway programs have been used for decades by academic institutions to improve diversity in medicine by increasing the representation of underrepresented groups in medicine (URiM) [1–6]. The Association of American Medical Colleges (AAMC) no longer defines URiM, allowing schools to define the term independently using their own context. Generally, URiM has referred to groups that have been historically excluded, but this definition has been fluid over the years. The United States physician workforce faces an urgent and ongoing need for diversity, which has been shown to improve outcomes of care, particularly for Black, Hispanic, and American Indian populations [7]. In 2019, 5% of physicians identified as Black, 5.8% of physicians identified as Hispanic, and 0.3% of physicians identified as American Indian [8]. To address these disparities, many academic institutions have adopted pathway programs to help bring students from these groups into the medical field.

While many studies evaluate pathway programs for undergraduate students and summer programs, we found a lack of longitudinal studies focused on high school students, their development, and long-term outcomes beyond graduation [9–13]. Gaps persist in understanding the specific barriers and facilitators that either help or hinder the trajectory of URiM high school students into the medical profession. Moreover, existing literature on pathway programs often focuses on quantitative outcomes such as enrollment or graduation rates, leading to a lack of qualitative data on the lived experiences of students in these programs. Therefore, we aimed to study the experiences and outcomes of a high school pipeline program targeted towards URiM students. As such, this study includes both long term quantitative outcomes data as well as qualitative data on the specific barriers and facilitators for students who participated in the DoT pipeline program during high school.

Social capital theory provided the conceptual lens through which we approached our study. This theory emphasizes how networks of relationships foster trust, engagement, and mutual support within communities. The University of Minnesota’s community-focused social capital model extends this framework by highlighting three networks: bonding, bridging, and linking [14, 15]. These three networks strengthen social connection and collective efficacy. Applying this lens allowed us to consider how social capital may influence URiM students’ sense of belonging and access to support within educational environments. This theoretical foundation guided our exploration of relationships, networks, and opportunities across contexts in the study.

This study aims to gain a deeper understanding of how early exposure to medicine through high school pathway programs, such as Doctors of Tomorrow (DoT), influences students’ long-term career development. In particular, we examine how these experiences influence persistence along the pre-medical track, the pursuit of medical degrees, as well as other healthcare professions.

## Materials and Methods

### Description of the Doctors of Tomorrow Program

The Doctors of Tomorrow (DoT) program was established in 2012 as a partnership between the University of Michigan Medical School and Cass Technical High School in Detroit [16–19]. DoT is designed to address disparities in healthcare by increasing the representation of URiM students. DoT works primarily with students from Cass Technical High School in Detroit, a large Title I school (a school that receives federal funding to assist students from economically disadvantaged backgrounds) with a predominantly racial and ethnic URiM student body of 98.1% [20]. More specifically, the student population at Cass Technical High is reflective of: 76.1% Black students, 11.7% Hispanic students, 9.4% Asian students, 1.9% White, and less than 1% American Indian or Pacific Islander students [20]. Additionally, Cass Tech High School has a graduation rate of 96%, well above the average rate of 64.5% in Detroit Public Schools Community District [20, 21]. From 2013 to 2019, the DoT students were comprised: 64.4% Black students, 9.7% Hispanic Students, 21.3% Asian students, 0.9% White students, 2.8% Mixed/Other, and 0.9% did not disclose [16].

DoT brings together physicians and medical students who mentor high school participants, facilitating early exposure to medical education, clinical shadowing, and hands-on research opportunities [22]. Additionally, DoT provides financial assistance to students participating in the program for items such as school supplies, participation in summer internships, and more. Through these initiatives, DoT seeks to contribute to the diversity of the healthcare workforce. Since its implementation, the program has graduated nine cohorts of students from Cass Technical High School and expanded to additional schools in the Detroit area, intending to create a sustainable pathway to careers in healthcare. Previously, DoT has been studied through its application process [16], curriculum [17], transition from high school to university [18], and summer internship program [19]. This study is the first time a long-term outcomes analysis has been conducted.

### Study Design

This study used a cross-sectional, survey-based design to assess the educational and career trajectories of DoT alumni. A literature review was conducted on prior outcomes studies for pipeline programs to identify gaps in the literature. DoT students and leadership collaborated to delineate information of interest during multiple program meetings and feedback sessions, developing the survey comprising 36 questions covering demographics, career goals, outcomes, and feedback. This quantitative survey with qualitative content analysis aimed to gather both quantitative and qualitative data on the barriers and facilitators alumni experienced in their pursuit of higher education and medical careers. Our study followed the Standards for Reporting Qualitative Research (SRQR) guidelines to create a comprehensive framework for qualitative methodological transparency [23]. Additionally, the study explored alumni’s undergraduate experiences, career paths, and the influence of the DoT program on their journey to medical school or other healthcare-related fields. The study was reviewed and considered exempt by the University of Michigan Institutional Review Board because it does not fit the definition of human subjects research.

### Study Population

The study population consisted of 184 alumni of the DoT program from the classes of 2016–2021. All participants were enrolled students in the program while attending Cass Technical High School in Detroit, Michigan. These students were identified as having an interest in pursuing careers in medicine and then selected for participation in DoT based on their placement in the chemical biological curriculum and desire to engage in healthcare-related experiences. No incentives were offered to participants.

### Data Collection

Data collection occurred from December 2022 to February 2023. Various methods were employed to contact alumni, including emails, phone numbers, and social media. The survey was administered via Qualtrics and consisted of 35 questions covering topics such as undergraduate major, college choice, pre-medical trajectory, work experiences, and barriers and facilitators to pursuing medical school. The full list of survey questions can be found in the Supplement. Reminders were sent at least once during the data collection period to increase response rates.

### Analysis

Quantitative data were analyzed using descriptive statistics to provide an overview of participants’ demographic information, educational trajectories, and career aspirations. Qualitative data from open-ended survey questions were analyzed using content analysis to identify common barriers and facilitators that participants encountered on their paths toward healthcare careers. Although the analytic framework was content analysis, it is informed by Braun and Clarke’s six-step approach to thematic analysis, which emphasizes familiarization with the data, generating codes, identifying themes, naming and defining themes, and writing a coherent narrative [25]. The process supported iterative and reflexive engagement with the data. The team comprised three previous DoT participants (ROH, DB, DT) alongside medical student mentors and administration. Two team members (BL, ROH) coded data inductively in Excel using in vivo codes to capture participants’ authentic language. Statistical methods for coding reliability were not used due to low sample size; instead, to ensure credibility, an independent audit was performed by each team member, and disparate codes were discussed and reconciled through team consensus to resolve interpretive differences. Therefore, all coding was agreed upon by the authors. Consistent with Malterud’s principle of information power, analysis continued until the data were richly and convincingly organized, with no new insights developing. Codes converged into themes of awareness, preparedness, and social capital, providing deeper insights into the experiences of DoT alumni.

## Results

### Quantitative Results

#### Participant Response and Demographics

Out of the 184 alumni contacted, we received responses from 75 participants, representing a 41% response rate. Of those who responded, 85% (64/75) provided demographic information. Among these, 61% (39/64) identified as African American, 29% (19/64) as Asian, 8% (5/64) as Latino/Hispanic/Spanish, and 2% (1/64) as White.

#### Higher Education

**Fig. 1:**
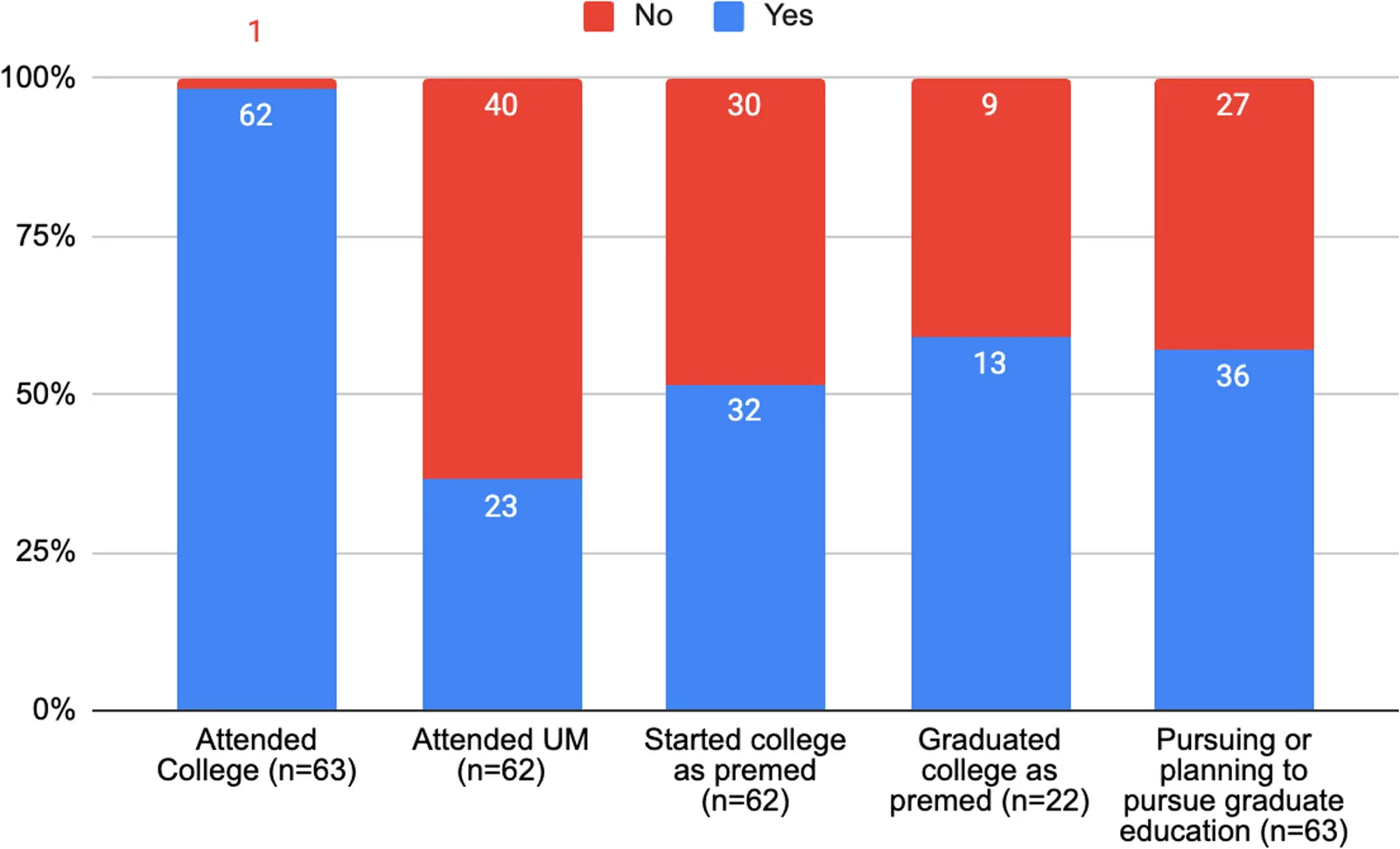
College attendance and projected trajectory. Out of the 63 completed responses with higher education data, 62 students attended college. Out of the 62 students who attended college, 23 attended the University of Michigan. Out of the 62 students who attended college, 32 students started college as pre-med. Out of the 22 students who have already graduated college, 13 graduated as pre-med. Out of the 63 completed responses, 36 students are pursuing or planning to pursue graduate education. Data were normalized to convey percentages

84% (63/75) of responses provided education data, which include college attendance, pre-medical trajectory, and plans for graduate education. These are summarized in Fig. 1. Nearly all participants (98%, 62/63) attended college, enrolling across 15 different institutions. Of those, 87% (54/62) attended colleges in Michigan, with 37% (23/62) of participants attending or having graduated from the University of Michigan and 32% (20/62) enrolled in Detroit-area institutions. Table 1 categorizes colleges attended by participants into three categories: University of Michigan, Universities Within the State of Michigan (excluding the University of Michigan), and All Universities Outside the State of Michigan.

**Table 1:** Colleges attended by survey respondents

Colleges Attended	Number (*n* = 62)
University of Michigan	23
Universities Within the State of Michigan (excluding the University of Michigan)	31
All Universities Outside the State of Michigan	8

**Fig. 2:**
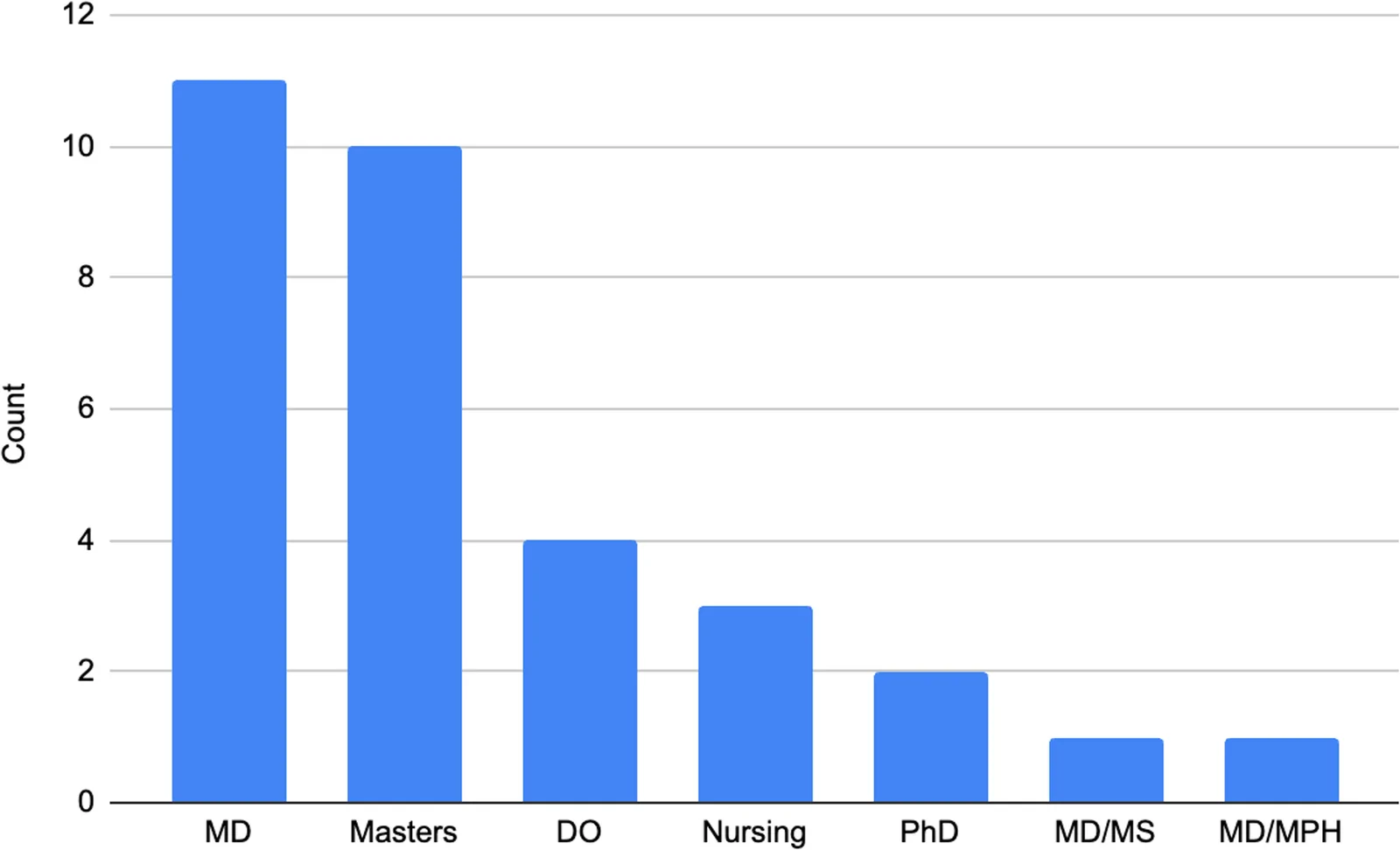
Anticipated/Desired graduate programs of DoT alumni (*n* = 32). Data shown from the 32 responses that answered the anticipated/desired graduate program question

A total of 52% (32/62) of respondents began college as pre-med, and 59% (13/22) completed their undergraduate studies on the pre-med track. Additionally, 57% (36/63) are currently pursuing or intending to pursue further education beyond their undergraduate degree. Of these, the majority (57%) aim to become physicians (MD, DO, or dual-degree), as illustrated in Fig. 2. Furthermore, many students expressed research interest, with 33% (10/30) intending to pursue a Master’s degree and 7% (2/30) aiming for a PhD. Notably, several respondents pursuing Master’s degrees viewed this as a strategic step to strengthen their medical school applications.

#### Work Experience

**Fig. 3:**
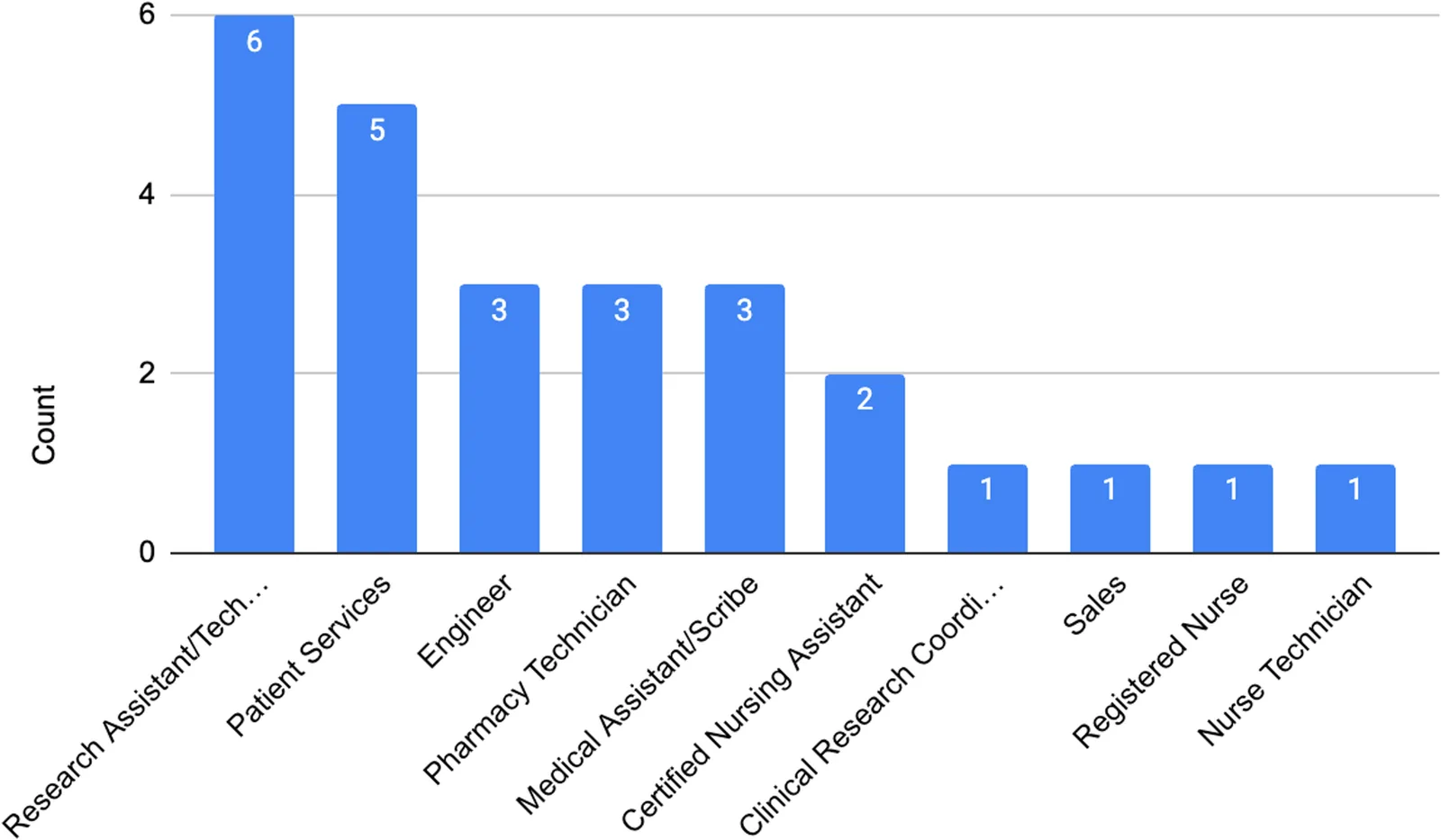
Healthcare work experience of DoT Alumni (*n* = 26). Patient services examples include medical assistant, nurse tech, resident aide. Data shown from the 26 responses included job title

DoT alumni reported gaining work experience in various healthcare and research roles (Fig. 3). 52% (31/60) respondents answered “yes” to the healthcare or STEM field question (29 answered “no”). Specifically, 28% (6/25) of participants were engaged in research, working as research assistants to strengthen their experiences for graduate school applications. Many others worked in patient-facing positions, including patient services (e.g. medical assistant, nurse technician, resident aide, 20%, 5/25), nursing (16%, 4/25), and as medical assistants (12%, 3/25). These roles provided valuable experience and furthered their career development in healthcare.

#### Educational Attainment

We found that 98% of respondents attended college, a rate higher than the overall college attendance rate for Cass Technical High School students (90%) [20]. Moreover, 37% of DoT alumni attended the University of Michigan, a selective public institution, compared to 8% of Cass Tech students overall [20]. These findings suggest that DoT alumni are not only more likely to attend college but also significantly more likely to attend a highly selective university like the University of Michigan.

#### Desire to Pursue a Career in Medicine

Although students join the DoT program as ninth graders, a slight majority (52%) pursued a pre-med track in college, despite exposure to other career paths in high school. Notably, the percentage of alumni who graduated on the pre-med track increased to 59%, suggesting that some students explore other fields early in their college careers but ultimately return to their interest in medicine.

Given that past studies show a significant drop-off in the number of students who remain pre-med through college, the high percentage of DoT alumni on the pre-med track is a notable success in that the program is retaining students’ interest in the medical field [24]. This persistence may be attributed to early exposure to healthcare through the DoT program. Alumni working in healthcare roles, conducting research, or pursuing advanced degrees further illustrate their commitment to becoming physicians. In fact, 53% (17/32) of respondents reported a desire to become physicians **(**Fig. 2**)**, indicating that the DoT program may have catalyzed sustained interest in medical careers.

### Qualitative Results

The qualitative data collected provides valuable insights into the barriers and facilitators that shaped students’ collegiate careers and interest in the medical field. The analysis revealed three main themes: awareness, preparedness, and social capital. Table 2 captures additional illustrative quotes from open-ended responses.

**Table 2:** Illustrative quotes from open-ended responses

THEME	DEFINITION	CODES	EXAMPLES
Awareness	The complete knowledge of the pre-medical process, programs, professions, and requirements.	Other professions, exposure, programs, interest, passion	“Through my scholarship program I found out I was more interested in the PhD track.” [Participant ID #33] “Lots of classes that I was not interested in. I am mainly interested in doing research in virology/immunology as of now.” [Participant ID #22] “A program where we would spend a semester during the summer here taking the intro classes and seeing how they are and possibly see if they could be taken for credit.” [Participant ID #22]
Preparedness	The state of being completely ready for college academically, financially, socially, and mentally.	Academics, time-management, finances, study, GPA	“Struggled passing classes Bio 172, Chem 215. Lost passion after failing.” [Participant ID #9] “Family care, and living environment.” [Participant ID #41] “The course load was too demanding for my work schedule. Imposter syndrome, too, probably.” [Participant ID #52]
Social Capital	Having peers and mentors that can aid you in success through a sense of community and resources.	Resources, community, network, support, mentorship	“Undergrad: introduced me to the various fields in medicine, the social determinants of health (I did my undergrad DoT capstone on mental health). Examining the healthcare system through a social science lens is something I am still interested in and I have taken coursework to increase my knowledge in the area. DoT set me up for success by giving me resources and community to decide my own future.” [Participant ID #52] “One thing I recommend is (if we have the resources) expanding DoT Succeed and communication to students at universities other than UMich!” [Participant ID #30] “Maybe host more community building events.” [Participant ID #32]

#### Awareness

Awareness refers to the level of understanding regarding the pre-medical process, available programs, various health professions, and their respective requirements. Students identified three areas where increased awareness could have enhanced their experience: knowledge of medical professions, institutions, and college majors.

Many students expressed that the DoT program seemed overly focused on training future medical doctors (MDs), excluding other healthcare professions such as dentistry, nursing, and physician assistant roles. Although this was not the program’s intention, the name and structure may have implied such exclusivity. The lack of focus on pathways like DO (Doctor of Osteopathic Medicine) further contributes to this perception. As a result, some students felt alienated from the program if they were interested in additional health professions. This could have been an opportunity to bridge networks for students to broaden their connections for additional opportunities. One participant shared:

*“I think if DoT included other medical professionals like dentist*,* optometrist*,* it would have introduced people to more medical professions that may have helped them in the long run.” [Participant ID #6]*.

Because the DoT program is housed and funded by the University of Michigan, students were predominantly informed about opportunities at this institution. While the program aimed to increase enrollment at the University of Michigan, students felt it limited their knowledge of other schools that might be a better fit socially, academically, or financially. Additionally, students wished for more awareness about post-baccalaureate programs and other pathways to medical school available at different institutions. This was elucidated by one participant:

*“The DoT program did a great job at exposing me to different medical careers and pathways. I wish that we had talked about different programs schools have like the med-direct program at Wayne State.” [Participant ID #23]*.

DoT has a strong focus on the educational components for pursuing a medical career. This is emphasized for high school and university courses. However, students expressed a need for increased awareness about the financial costs associated with medical school, as well as the financial assistance available. One of the participants explained:

*“Doctors of Tomorrow has a great way to expose students to the field and help them get into medical school but DoT can improve by assisting on a financial aspect*,* for example*,* ensuring tuition-free medical school for DoT students.” [Participant ID #18]*.

#### Preparedness

Preparedness is defined as being fully ready for college—academically, financially, socially, and mentally. While academic resources are often seen as a primary challenge for underrepresented students, financial and social factors emerged as significant barriers.

Financial challenges were consistently highlighted. Many students struggled with the high cost of living in Ann Arbor, where the University of Michigan is located, and took on multiple jobs to support themselves and their families. Mentors in the DoT program often linked students to resources within the program and on campus to support each student’s success. Balancing work, family responsibilities, and academics was a significant strain, leaving students with difficult choices between their academic goals and family obligations. Two illustrative quotes from participants reflect this tension:

*“Finances are one of the biggest challenges with pursuing pre-med.” [Participant ID #20]*.

*“At the moment*,* it’s just the time commitment and finding a way to study for the MCAT.” [Participant ID #26]*.

Many students from Cass Technical High School faced gaps in their high school education, such as a lack of adequate staffing, which created challenges in college science, technology, engineering, and mathematics (STEM) classes. These knowledge gaps, combined with the pressure to maintain a high grade point average (GPA), led some students to switch out of pre-med tracks. One participant described this experience as follows:

*“I went without teachers in chemistry*,* geometry*,* algebra*,* and biology in high school. I never got the education I needed for STEM classes in college*,* so I gave up the pre-med track to save my GPA.” [Participant ID #42]*.

#### Social Capital

Social capital refers to the network of peers, mentors, and resources that can support students’ success. In medicine, having a strong support system is critical, particularly for underrepresented students. The DoT program fostered a sense of community for students who attended the University of Michigan, as they had their cohort for support. By bonding networks, students could experience a close connection to feel a sense of belonging and continue their journey to medicine. However, students who attended other institutions often felt a lack of community and support. One of the participants explained:

“*I think the DOT program was a great help with supporting the pre-med path but I just realized that it wasn’t for me. I think I would have continued a pre-med track if I were to have gone to U of M but since I was not accepted and no longer have that support system around me*,* I wasn’t as influenced to be in the pre-med field anymore.” [Participant ID #28]*.

The DoT mentorship component pairs ninth-grade students with first-year medical students, with the idea that mentors and mentees would eventually graduate together. While this mentorship provided a connection to the medical field, the strength of the relationship varied with communication and scheduling with medical student mentors. Some students felt the mentorship could have been more structured and focused. One participant shared:

*“I feel as though the Doctors of Tomorrow program did a great job at preparing me. I do believe that mentorship could have been a lot stronger.” [Participant #50]*.

Regardless of the institution they attended, a strong social network is crucial for URiM students in medicine [26]. Many participants emphasized the importance of networking with peers, faculty, and professionals. Those who did not attend the University of Michigan expressed a desire for continued engagement with the DoT community to maintain those important connections. This challenge was described by one of the participants:

*“Because I didn’t continue with the program due to moving*,* I fell off a bit my junior/senior year which led to me ending up not following down the medical path fully. Maybe more engagement with members of the program that have left Cass and still inviting them to events and resources” [Participant ID #52]*.

## Discussion

Our study highlights that there is no “one-size-fits-all” solution for increasing the representation of underrepresented students in medicine. We identified specific barriers related to time management, finances, and academics, which we categorized into three key areas: awareness, preparedness, and social capital. Although these themes have been discussed before individually, our findings showcase how pipeline programs can collectively address these themes for better outcomes. Based on these findings, programs aimed at supporting URiM students are strengthened by: (1) use of inclusive language (e.g. outreach programs), (2) ensuring students are fully informed about opportunities that support their success, and (3) emphasizing the importance of a robust social network that can support students throughout their medical careers. As such, our results provide a generalized framework in which pipeline programs that aim to increase URiM representation can better address the needs of their students. By targeting these areas, we hope to increase the number of successful underrepresented students pursuing medical careers.

Pathway programs offer structured yet flexible opportunities for URiM students to navigate individualized educational experiences while pursuing the shared goal of medical school matriculation. The themes of awareness, preparedness, and social capital in action align with the University of Minnesota’s community-focused social capital model, which emphasizes the role of connection, engagement, and trust in fostering academic and professional development [14, 15]. This theoretical alignment illustrates how student-identified needs can be understood within a broader framework of social capital development. It also highlights the corresponding actions that mentors and programs can take to ensure students receive the relational and institutional support necessary for success.

Awareness must encompass various medical professions, programs at different institutions, and the specific requirements for medical school matriculation. Pre-exposure programs need to use more inclusive language to remind students that they are on a flexible pathway. Although students can research programs independently, studies have shown that many pathway programs have an insufficient online presence or lack adequate information, creating an early barrier to entry [27]. For example, students who pursued Master’s programs to prepare for medical school may have considered postbaccalaureate programs had they been aware of these options. Supplying students with relevant information serves as a powerful catalyst for success, instilling the confidence and preparedness necessary to select and pursue their ideal career trajectory in healthcare.

Preparedness for a career in medicine goes beyond academic readiness—it requires mental, social, and financial preparation. Students attending predominantly white institutions (PWIs), in particular, need to be prepared for potential social shifts, as they may be among a small number of URiM students on campus [28]. College preparedness starts early in high school, shaped by factors such as class selection, financial readiness, and the relationship students have with their high school counselors [29]. These early experiences play a critical role in setting students up for success. Pathway programs geared towards underrepresented students could offer more robust resources to help students overcome financial barriers. Examples include stipends or work-study programs to support students with limited incomes.

Finally, a strong social network, conceptualized as social capital, is a critical asset in the medical field. The development of this capital begins with intentional mentorship and structured support during high school, guided by frameworks that prioritize time, resources, and ongoing support [30]. Many students viewed the University of Michigan as an appealing choice due to the community support provided by current college students and alumni of DoT. On campus, mentorship, study groups, and academic resources fostered connection and belonging, which may be unfamiliar at other institutions. Consistent with other pre-health pathway programs, strong mentorship served as a key factor linking students to healthcare opportunities and promoting long-term success [31].

### Limitations and Future Research

Our study had limitations worth noting. The response rate of 41% falls within the typical range for surveys assessing outcomes in similar pathway programs. However, this rate, combined with the relatively small cohort sizes, made year-by-year analysis difficult and limited our ability to gather comprehensive quantitative and qualitative data. Additionally, many respondents did not complete the optional sections of the survey, resulting in some response samples being as low as 20 participants. This introduces potential response bias, as the survey may have disproportionately attracted alumni who are more successful or those who remain interested in healthcare careers, possibly overrepresenting those still pursuing the program’s goals six years later. Notably, the study sample has a large proportion of Asian students (29%, 19/64) compared to the overall makeup of Cass Technical High School (9.4%), as well as the 2013–2019 DoT cohorts (21.3%) [16]. As such, both the study sample and the DoT population may differ from the overall Cass Technical High School student body. Therefore, limitations related to response bias and small subgroup samples impact the interpretation of our findings. Future studies with sufficient sample sizes could further analyze differences in subgroups.

Additionally, the retrospective nature of the survey could cause potential recall bias within the study design. Our data did not collect the number of students who wanted to be physicians, independent of the pathway program. Future studies will need to expand to participants at additional high schools with programs similar to DoT to validate results across contexts. This would allow study findings to be more generalizable and transferable to other underserved populations.

Despite substantial investment in the DoT program to support aspiring physicians, DoT has not yet seen measurable matriculation outcomes, suggesting substantial gaps along the pathway. Nevertheless, we find key factors such as awareness, preparedness, and social capital that shape students’ persistence in their aspirations towards medical school. Our findings indicate that awareness fosters motivation, preparedness builds confidence, and social capital provides access and opportunities. Cultivating this synergy may help pathway programs enhance their support and guidance of students.

## Data Availability

De-identified raw survey results are available upon reasonable request.
